# Review of the role of dynamic 18F-NaF PET in diagnosing and distinguishing between septic and aseptic loosening in hip prosthesis

**DOI:** 10.1186/s13018-014-0147-7

**Published:** 2015-01-16

**Authors:** Olu Adesanya, Andrew Sprowson, James Masters, Charles Hutchinson

**Affiliations:** University of Warwick, Coventry, CV4 7AL England UK; University Hospital Coventry and Warwickshire, Coventry, CV2 2DX England UK

**Keywords:** F-18 fluoride PET, Arthroplasty, Joint prosthesis, Septic loosening, Aseptic loosening

## Abstract

Joint replacements may fail due to infection, dislocation, peri-prosthetic fracture and loosening. Between 0.4 and 4% of joint replacements are known to be complicated by infection and aseptic loosening 2–18%. Differentiating between infection and aseptic loosening has an important bearing on the ongoing strategy for antimicrobial therapy and surgical intervention, but distinguishing one from the other can be difficult and will often require a battery of clinical and biochemical tests including the use of varying radiological modalities to accurately identify whether problematic joints are infected or aseptically loose. Prompt diagnosis is important due to the development of a biofilm on the surface of the infected prosthesis, which makes treatment difficult. There is no consensus among experts on the ideal imaging technique nor the methodology for image interpretation, but there is an increasing trend to apply hybrid imaging in the investigation of painful joint prosthesis and recent attempts have been made using PET-CT to identify aseptic loosening and infection with ^18^F-fluorodeoxyglucose (FDG) and sodium fluoride ^18^F-Na. The aim of this paper is to evaluate the role of ^18^F-NaF sodium fluoride (^18^F-NaF) positron emission tomography (PET) in distinguishing between septic and aseptic failure in hip and knee replacements, in addition to evaluating the feasibility of using multi-sequential ^18^F-NaF PET-CT for the assessment of painful lower limb prostheses.

## Background

Joint replacement is the surgical intervention of choice for end-stage arthritis. A small but significant proportion of these patients with joint prostheses go on to suffer implant failure. Causes for failure include infection, dislocation, peri-prosthetic fracture and loosening.

It is estimated that between 0.4 and 4% of joint replacements are known to be complicated by infection [1] and aseptic loosening 2–18% [2]. Although differentiating between infection and aseptic loosening has an important bearing on the ongoing strategy for antimicrobial therapy and surgical intervention [3], distinguishing one from the other can be difficult and will often require a battery of clinical and biochemical tests including the use of varying radiological modalities to accurately identify whether problematic joints are infected or aseptically loose. Prompt diagnosis is important due to the development of a biofilm on the surface of the infected prosthesis, which makes treatment difficult [4].

There is currently no consensus among experts on the ideal imaging technique nor the methodology for image interpretation [5]. Current imaging investigations used in problematic prosthetic joints include serial radiographs, contrast arthrography, ultrasound, magnetic resonance imaging (MRI), computed tomography (CT) and conventional Nuclear Medicine studies [6], supported by blood tests such as erythrocyte sedimentation rate (ESR), C-reactive protein (CRP) and the white cell count [7]. In the absence of other inflammatory conditions, the white cell count is least useful [8], while CRP is most useful from the third week after joint replacement surgery, but ESR may take 3 to 12 months to return to normal levels [7,8].

CT and MRI imaging for prostheses produce high anatomical detail, but metal-related artefacts can reduce image quality and sensitivity. Ultrasound can detect soft-tissue collections; and help guide joint aspiration and capsular biopsy. In general, radionuclide imaging techniques yield little anatomical detail and are sensitive but non-specific for diagnosing loosening and infection.

Thus, there is an increasing trend to apply hybrid imaging in the investigation of painful joint prosthesis, often employing SPECT-CT, which combines the high resolution of CT and the functional sensitivity of Single Photon Emission Tomography (SPECT) [9]. Recent attempts have been made using PET-CT to identify aseptic loosening and infection with ^18^F-fluorodeoxyglucose (FDG) and sodium fluoride ^18^F-NaF [10,11]. Sodium fluoride metabolism in bone is reliant on the rate of blood flow, which is the rate-limiting step. Most of the sodium fluoride delivered to the bone is retained after a single pass of blood [12] making it an excellent radiopharmaceutical to assess subtle changes in bone hyperaemia and turnover [13]. The first pass rate varies among different bone types [12], with the degree NaF uptake in bone marrow being negligible when compared with the bony cortex levels [12]. ^18^F-NaF bone uptake is dependent on the exchange of fluoride ions with hydroxyl ions in hydroxyapatite crystal to form fluoroapatite [13]. ^18^F-NaF is freely diffusible across membranes and 1 h after injection, only 10% of ^18^F-NaF remains in the plasma. ^18^F-NaF is rapidly cleared from plasma and excreted by the renal system following glomerular filtration and tubular secretion [12,14].

The aim of this paper is to evaluate the role of ^18^F-NaF sodium fluoride (^18^F-NaF) positron emission tomography (PET) in distinguishing between septic and aseptic failure in hip and knee replacements, in addition to evaluating the feasibility of using multi-sequential ^18^F-NaF PET-CT for the assessment of painful lower limb prostheses.

## Methodology

### Study design

A systematic review of the literature was undertaken according to the methods described in the Cochrane Handbook for Systematic Review of Interventions.

### Research question

What is the evidence for use of sodium fluoride PET-CT in differentiating between septic and aseptic failure of hip and knee replacements?

### Inclusion criteria

Human prospective studies that reported data on sodium fluoride in joint prosthesis imaging to diagnose loosening and/or infection.

### Exclusion criteria

Studies were excluded where other isotopes other than sodium fluoride were used. Studies were limited to English language and filters for human studies and clinical trials were applied.

### Search strategy

Studies were identified using MeSH terms and keywords in MEDLINE, EMBASE, Cochrane and Dynamed. About 133 were excluded at title and abstract; 1 was excluded at full paper review. No paper was added after review of the references.

PubMed MESH search terms -(((((((((infection) OR sepsis) OR loosening) OR aseptic loosening) OR osteolysis)) AND ((((prosthesis) OR joint replacement) OR knee replacement) OR hip replacement)) AND ((((((sodium fluoride) OR fluoride) OR fluorine) OR NaF) NOT FDG) NOT fluorodeoxyglucose))) NOT dental.

Two authors checked all data used in the analysis. When disagreements arose, these were resolved by consensus.

Initially, title and abstracts were reviewed. Potentially relevant papers were reviewed in their entirety. The references cited by each potentially relevant paper were scrutinised in order to locate additional potentially relevant papers.

### Statistics

The summative weighted sensitivities and specificities were calculated from the data extracted.

## Results

Following data extraction, three studies were selected which satisfied the required characteristics. Data were extracted from each study and are summarised in Table 1.

**Table 1:** **Summary of included studies**

**Year**	**Authors**	**Patients**	**Gender (M/F)**	**Hip joints**	**Knee joints**	**Symptomatic joints**	**Control group**	**Asymptomatic control joints**	**Minimal time from surgery**	**Minimal clinical follow-up**	**Surgical follow-up**	**Differentiate aseptic loosening from sepsis**
1976	Creutzig H. et al.	31	N/A	31	0	13	Yes	18	3 to 9 months	12 months	5	No
2007	Sterner et al.	14	9/6	0	14	14	No	0	1.1 years	6 months	6	No
2011	Kobayashi N et al.	49	N/A	65	0	38	Yes	27	1 year	12 months	11	Yes

The review identified three prospective studies [15-17] that met our search criteria. The selected studies consist of a total number of 94 patients which looked at 110 joints. There were 96 hips and 14 knees, of which 35 were asymptomatic and 65 joints were symptomatic. Only one study differentiated aseptic loosening from infection [15]. Minimal time from surgery varied from 3 months to just over 12 months. Twenty-two patients were followed up surgically, while the remaining patients were followed up clinically for periods varying from 6 to 12 months. A weighted average of sensitivity and specificity of the different studies was determined from the three studies. The sensitivity of ^18^F-NaF-PET in identifying prosthesis infections was found to be 97.04%; this was calculated from the weighted average sensitivity from the three different studies. The weighted specificity, PPV, NPV and accuracy are 88.11, 84.68, 98.82 and 87.33%, respectively. Of the three studies, Sterner et al [16] reported the lowest specificity. These results are reported in Table 2.

**Table 2:** **Results from the included studies**

**Authors**	**Patients (joints)**	**TP**	**TN**	**FP**	**FN**	**Sensitivity**	**Specificity**	**PPV**	**NPV**	**Accuracy**
Creutzig H. et al.	31 (31)	13	14	4	0	100	0.78	0.76	100	0.87
Sterner et al.	14 (14)	5	5	4	0	100	0.56	0.56	100	0.71
Kobayashi N et al.	49 (65)	36	23	6	2	0.95	0.88	0.95	0.98	0.91

**Figure 1:**
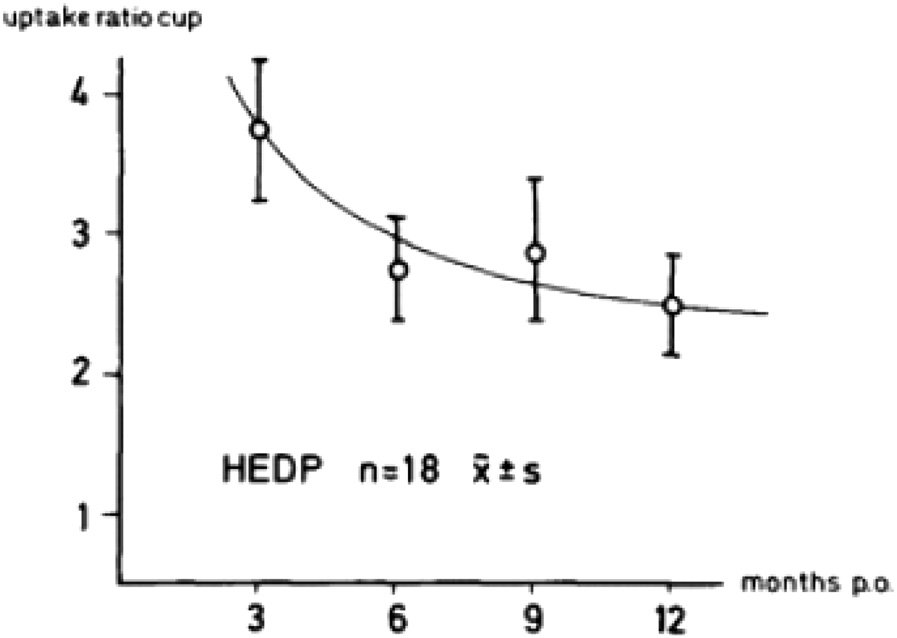
**Uptake ratios in patients without complications over the cup.**

**Figure 2:**
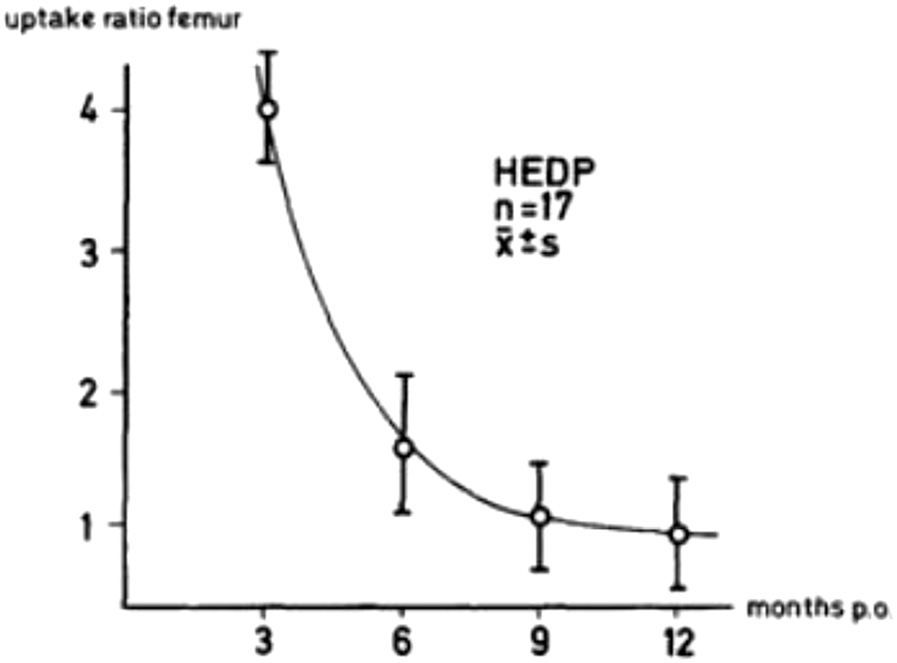
**Uptake ratios in patients without complications over the thigh.** Graphical representation of normal HEDP uptake levels in the acetabular (Figure 1) and femoral components (Figure 2). NaF uptake levels are said to be similar (14). Reproduced from Creutzig H. Bone imaging after total replacement arthroplasty of the hip joint. A follow-up with different radiopharmaceuticals. Eur J Nucl Med. 1976 Aug 12;1 (3):178 (Figures 1 and 2) with kind written permission from Springer Science + Business Media B.V.

The value of fluoride in bone imaging was first recognised by Monte Blau and team in 1962 [18,19], but Hans Creutzig in Hannover was the first to demonstrate the importance of ^18^F-NaF-PET in joint prosthesis infection in 1976 with 31 total hip replacements [17]. He also mapped the comparable normal peri-prosthetic pattern of both Tc^99m^ HEDP (hydroxyethylidenediphosphonate) and ^18^F-NaF uptake in the post-surgical period from 3 to 12 months using uptake ratio in both symptomatic and asymptomatic prostheses. The ratios were found to decline rapidly (Figures 1 and 2) and reach a nadir at 6–9 months following surgery with more rapid decline demonstrated in the femoral component [17]. Interestingly, departures from this normal pattern of decline preceded the development of symptoms in patients with bone and soft tissue infections [17]. In this study, the distinction between aseptic loosening and infection was unclear. Images were acquired 3 h after the injection of 148 MBq of ^18^F sodium fluoride. Relatively poor quality images from using coincidence imaging and a 5″-rectilinear scanner may have accounted for inability to distinguish soft tissue infection from bone infection [17]. The 3-h uptake period would also have been detrimental because early high target-to-background ratios in result in peak bone uptake levels around 45–60 min after radioisotope injection [20]. At 60 min after radioisotope injection, only 10% of F18- fluoride remains in the plasma due to negligible plasma protein binding, rapid blood and renal clearance, and high bone uptake [20].

Thomas Sterner performed the only prospective study with the use of ^18^F-NaF in imaging knee prosthesis, but no attempt was made to differentiate aseptic loosening from infection [16]. Fourteen symptomatic knee prostheses were examined and no control group was employed. Of these 14 patients, 6 underwent surgery for confirmation of the imaging findings and the other 8 were followed up clinically for 6 months. The relatively low specificity in this study may have arisen from several factors including the fact that this the study included only knees, the sample size was relatively small and finally that even intermediate levels of peri-prosthetic uptake were regarded as positive for aseptic loosening or infection [16]. He also regarded the scan as abnormal if (a) there was more uptake in the prosthesis/bone interface than in normal/bone/soft tissue or contralateral asymptomatic prosthesis, (b) the increased uptake included half the bone/metal interface in the femoral component or (c) if the tibial stem in the tibial component was involved. Furthermore, they discovered that the use of semi-quantitative analysis with standardised uptake values (SUVs) yielded no added value [16]. Image quality in this study was bound to be better because the authors employed an ECAT-Exact HR+ (Siemens Medical Systems) PET scanner 1 and 60 min after the injection of 350 MBq of^18^F fluoride [16].

In 2011, Naomi Kobayashi et al. published a prospective study of ^18^F-NaF PET in 65 hip prostheses; to date, this has been the only prospective trial which differentiated aseptic loosening from sepsis [15]. Images were acquired 40 min after injection of 185 MBq of ^18^F fluoride using a SET 2400W device (Shimadzu, Kyoto, Japan). She was able to distinguish between normal, aseptic loosening and infected prostheses. The first method involved measuring the degree of ^18^F fluoride uptake in peri-prosthetic tissues (SUVmax). Average values for the normal, aseptic and septic loosening prostheses were estimated to be 4.9 ± 2.5, 8.1 ± 2.9 and 10.5 ± 3.4, respectively [15]. When a threshold SUVmax of 6.9 is applied for diagnosis of infection, the test had a sensitivity and specificity of 81 and 80%, respectively [15]. Furthermore, when a threshold SUVmax of 4.9 is applied for aseptic loosening, the test yields a sensitivity and specificity of 95 and 82%, respectively [15].

PET images were also analysed for the pattern and distribution of ^18^F fluoride PET uptake and categorised into three types. Type 1 uptake showed no significant ^18^F fluoride uptake; type 2 uptake shows mild localised uptake on the cup side or stem. Type 3 pattern of uptake demonstrates significant uptake which extends through more than half of the bone-implant interface [15]. In type1 pattern, 96% of the cases were normal, 80% of type 2 pattern were due to aseptic loosening and 95% of type 3 pattern cases were due to infection [15]. The final diagnosis of infection was obtained by a combination of microbiologic culture, histopathology and polymerase chain reaction (PCR) analysis of bacterial DNA with two different primer and probe sets, one specific for the detection of methicillin-resistant staphylococcus and another for broad-range detection by universal PCR that targets a part of 16S rDNA gene, with increased sensitivity [15,20]. Many PCRs that detect the universal 16S rRNA bacterial gene have problems with false-positive results, with necrotic bacteria detected by PCRs [21]. The clinical importance of positive results in the absence of other clinic-pathologic and radiological features of infection is of uncertain significance, but specificity can be improved by combining a universal PCR with subsequent bacterial sequencing [21].

## Discussion

It is important to distinguish between infection and loosening because the management of these conditions is very different. There is no consensus on the use of imaging algorithms for the diagnosis of loosening and infection. In general, radionuclide imaging techniques are sensitive but non-specific for diagnosing loosening and yield little anatomical detail. Furthermore, a number of radionuclide studies are often performed sequentially to improve diagnostic accuracy [22], further prolonging the interval between the onset of symptoms and diagnosis [23]. Sodium fluoride positron emission tomography (^18^F-NaF-PET) is a promising tool with high sensitivity and specificity in the assessment of joint replacements, but it possibly will be of limited use before the ninth post-surgical month when the uptake curve ratios reach a nadir particularly in the acetabular component [14]. This can be overcome by routinely imaging ‘at risk’ prosthesis at 3-month intervals for the detection of abnormal rates of decline in peri-prosthetic ^18^F-NaF uptake. The downside of this method would be its relatively high cost and high radiation dose [3], but with reduced scanning times with PET-CT [24], this may become a more attractive option. ^18^F-NaF-PET also avoids patient contamination as well as infection risk to technicians which may occur in labelled white cell studies. The added value of the CT component in ^18^F-NaF PET-CT would include the detection of anatomical lesions such as occult fractures, aseptic loosening and osteolysis as well as guiding intervention procedures [6].

Alternately, with fluorodeoxyglucose positron emission tomography (^18^F-FDG-PET), the most commonly used PET isotope, arthroplasty induces non-specific periprosthetic increased uptake which persists for several years, even in patients without evidence of infection or loosening [25].This can easily be misinterpreted as periprosthetic infection resulting in false positives most notably in the head and neck portions of femoral prostheses [25].

## Conclusion

Serial or single sodium fluoride positron emission tomography (^18^F-NaF-PET) is a sensitive and specific tool for assessing joint prostheses. Furthermore, the CT component enhances its diagnostic and radiological interventional value. Wider studies are required to identify normal and pathological patterns in both hip and knee prostheses.
